# Genomic Tools for the Identification of Loci Associated with Facial Eczema in New Zealand Sheep

**DOI:** 10.3390/genes12101560

**Published:** 2021-09-30

**Authors:** Kathryn M. McRae, Suzanne J. Rowe, Patricia L. Johnson, Hayley J. Baird, Neil G. Cullen, Matthew J. Bixley, Jeffrey E. Plowman, Santanu Deb-Choudhury, Rudiger Brauning, Neville C. Amyes, Ken G. Dodds, Sheryl-Anne N. Newman, John C. McEwan, Shannon M. Clarke

**Affiliations:** 1 AgResearch Limited, Invermay Agricultural Centre, Puddle Alley, Mosgiel, Private Bag 50034, New Zealand; Suzanne.Rowe@agresearch.co.nz (S.J.R.); Tricia.Johnson@agresearch.co.nz (P.L.J.); Hayley.Baird@agresearch.co.nz (H.J.B.); Matt.Bixley@nesi.org.nz (M.J.B.); Rudiger.Brauning@agresearch.co.nz (R.B.); Ken.Dodds@agresearch.co.nz (K.G.D.); John.McEwan@agresearch.co.nz (J.C.M.); Shannon.Clarke@agresearch.co.nz (S.M.C.); 2 AgResearch Limited Ruakura Agricultural Centre, Bisley Road, Hamilton 3214, New Zealand; Neil.Cullen@agresearch.co.nz (N.G.C.); Neville.Amyes@agresearch.co.nz (N.C.A.); 3 AgResearch Limited, Lincoln Research Centre, Springs Road, Lincoln, Private Bag 4749, New Zealand; Jeff.Plowman@agresearch.co.nz (J.E.P.); Santanu.Deb-choudhury@agresearch.co.nz (S.D.-C.); Sheryl-Anne.Newman@agresearch.co.nz (S.-A.N.N.)

**Keywords:** sheep, disease, facial eczema, haemoglobin, GWAS

## Abstract

Facial eczema (FE) is a significant metabolic disease that affects New Zealand ruminants. Ingestion of the mycotoxin sporidesmin leads to liver and bile duct damage, which can result in photosensitisation, reduced productivity and death. Strategies used to manage the incidence and severity of the disease include breeding. In sheep, there is considerable genetic variation in the response to FE. A commercial testing program is available for ram breeders who aim to increase tolerance, determined by the concentration of the serum enzyme, gamma-glutamyltransferase 21 days after a measured sporidesmin challenge (GGT21). Genome-wide association studies were carried out to determine regions of the genome associated with GGT21. Two regions on chromosomes 15 and 24 are reported, which explain 5% and 1% of the phenotypic variance in the response to FE, respectively. The region on chromosome 15 contains the β-globin locus. Of the significant SNPs in the region, one is a missense variant within the haemoglobin subunit β (*HBB*) gene. Mass spectrometry of haemoglobin from animals with differing genotypes at this locus indicated that genotypes are associated with different forms of adult β-globin. Haemoglobin haplotypes have previously been associated with variation in several health-related traits in sheep and warrant further investigation regarding their role in tolerance to FE in sheep. We show a strategic approach to the identification of regions of importance for commercial breeding programs with a combination of discovery, statistical and biological validation. This study highlights the power of using increased density genotyping for the identification of influential genomic regions, combined with subsequent inclusion on lower density genotyping platforms.

## 1. Introduction

Facial eczema (FE) is a metabolic disease of major welfare concern and economic loss to farmers in New Zealand. The disease is caused by ingestion of the mycotoxin sporidesmin, which is found in the spores of the fungus *Pseudopithomyces chartarum* (formerly *Pithomyces chartarum*) [1,2]. *Pse. chartarum* grows on the dead leaf matter at the base of pastures, and ingestion of the toxic fungal spores by ruminants leads to liver inflammation and bile duct blockage [3]. While in many cases, animals might show no clinical signs of disease [4], production losses arise from a decrease in production [5] and lowered reproduction [6,7,8] in both clinically and sub-clinically affected animals. In an outbreak in 1981, the production cost to New Zealand was estimated to be NZD 266 million (inflation-adjusted to 2019 [9,10]). Clinical FE is characterised by photosensitivity in exposed areas of the animal, particularly in lightly pigmented areas such as the face, giving the disease its name [11]. Although FE outbreaks have been the most severe in the North Island of New Zealand [1], outbreaks have also been recorded in Australia [12], South Africa [13], North and South America [14,15], Europe [16] and China [17]. Outbreaks are associated with increases in dead leaf matter, temperature, and humidity in the summer and autumn months [18,19,20], and long-term climate change projections indicate that there will be an increase in the geographical spread of *Pse*. *chartarum* in New Zealand, resulting in outbreaks of FE in currently unaffected regions [21]. 

Current strategies for preventing the severity of FE include protecting animals through dosing with zinc compounds [22], spraying pasture with fungicide [23], pasture management or alternative feeds [24], and breeding animals for tolerance [25]. Initial research in the 1970s involved challenging animals with a measured dose of sporidesmin to assess the physiological impact. Post-challenge, scoring liver damage showed between-animal variation in susceptibility to FE, with a moderate to high heritability (h^2^) of 0.42 ± 0.09 [26]. Selection lines using Romney sheep were subsequently established in 1975 and were initially selected for tolerance or susceptibility based on liver damage [26]. A live-animal phenotype was reported in 1978 by Towers and Stratton [27], who showed that serum γ-glutamyltransferase (GGT) collected 2 to 3 weeks after sporidesmin challenge could be used as a measure of liver damage. Using data from the above selection lines, the heritability of log-transformed serum GGT levels at 21 days after a measured sporidesmin challenge (GGT21) was 0.45 ± 0.03 [28]. A commercial testing programme, Ramguard™, was then developed in the mid-1980s to provide New Zealand sheep breeders with sporidesmin and an ethical dosing strategy for ascertaining FE tolerance [29]. The resulting GGT21 values are used to generate breeding value estimates for tested and related animals, with 800 to 1100 rams tested each year. Recent re-estimation of genetic parameters for FE tolerance using a Ramguard™ dataset found a comparable heritability with that estimated from the selection lines (0.44 ± 0.03), with no significant genetic correlations between GGT21 and any production traits [30].

Several approaches have been used to search for causative genes or loci underlying FE tolerance, including a candidate gene approach [31,32], genome-wide scans for quantitative trait loci (QTL) [33,34] and scanning for selective sweep signatures [35]. There was limited concordance between the results of the studies, with only two of the selective sweep regions, on chromosomes OAR1 and OAR13, found within suggestive (F-statistic value > 10.2) QTL regions. The development of single nucleotide polymorphism (SNP) arrays (www.sheephapmap.org), facilitated significant increases in the number of genotyped animals, driving the development of genomic selection [36]. As a result, the number of animals with SNP genotypes and FE phenotypic measurements (GGT21) has increased. 

The objective of our study was to determine genomic variants associated with FE in New Zealand sheep, through a combination of discovery, statistical and biological validation using cost-effective lower density genotyping platforms. 

## 2. Materials and Methods

### 2.1. Animals and Genotyping

Sheep were selected for inclusion based on GGT21 records and genotype availability. All animals were from either industry or progeny test flocks and had been tested through the Ramguard™ programme. All data were obtained from the Sheep Improvement Limited (SIL, www.sil.co.nz) database. The Ramguard™ programme [29] uses *Pse*. *chartarum* that is cultured in a laboratory to produce the mycotoxin sporidesmin. Animals are dosed with precise amounts of sporidesmin (made up of toxic forms of sporidesmin, specifically sporidesmin A, B and E, with a predominance (>90%) of sporidesmin A) by intra-ruminal intubation at a volume that is dependent on the animal’s live weight (mg per kg live weight). Blood samples are taken for GGT testing before dosing (for a base level) and at 21 days after dosing and are processed through a commercial analytical laboratory. 

The initial dataset consisted of 2669 animals with GGT21 records from 45 flocks, born between 1992 and 2012. Animals were either one of the main dual-purpose sheep breeds used in New Zealand, including Romney, Coopworth and Perendale, or a composite of dual-purpose breeds. Individuals had been genotyped with either the Ovine Infinium^®^ HD SNP BeadChip (*n* = 137 animals; 606,006 makers; “HD”) or the Illumina OvineSNP50 BeadChip (*n* = 2532 animals; 53,903 markers; “50K”) according to the manufacturer’s protocol. The 2532 animals genotyped using the 50K chip were a subset of the animals used to develop genomic selection for FE, as described in Phua et al. [36].

SNPs that reached a genome-wide significance threshold (*p* < 1.04 × 10^−7^) from the initial ‘discovery’ analyses (Table S1) were subsequently included in the design of the Illumina OvineLD BeadChip (15,000 markers; “15K”). A subsequent ‘validation’ dataset consisted of 2363 animals with GGT21 records from 42 flocks, born between 2010 and 2016. As above, animals were either one of the main dual-purpose sheep breeds used in New Zealand or a composite breed. All validation animals were genotyped with the 15K chip according to the manufacturer’s protocol. 

Genome coordinates for all SNP were based on the OAR_v3.1 ovine genome assembly (GenBank assembly accession GCA_000298735.1 [37]). Quality control (QC) checks excluded markers that appeared non-autosomal (including pseudo-autosomal), had a call rate below 95%, and/or had a minor allele frequency (MAF) ≤ 0.05. Individuals were excluded from the analysis if there was more than 5% genotyping failure. After marker and sample QC, 2669 phenotyped animals were available for the discovery analysis, with 481,400 of the 606,006 markers utilised. For the validation dataset, 2342 phenotyped animals were available, with 14,747 of the 15,000 markers utilised for analysis. 

### 2.2. Imputation

For the 2532 animals genotyped using the 50K chip, imputation from 50K to HD genotypes for the discovery analyses was performed using Fimpute (v2.0) [38]. The imputation dataset included animals that did not have GGT21 records and consisted of 12,945 animals genotyped with the 50K chip, and 2408 animals genotyped with the HD chip. Quality control was carried out before imputation, with markers excluded that appeared non-autosomal (including pseudo-autosomal), had a call rate below 95%, had >2% reproducibility between platforms, and/or had >2% Mendelian errors. Animals included in the imputation dataset were born between 1965 and 2012, and represented 236 flocks, with the largest flock contributing 124 animals. Purebred Romney (*n* = 616), Perendale (*n* = 207) and Primera (*n* = 149) genotypes were used to calculate breed-specific imputation accuracies. Imputation accuracy was calculated using sets of 100 animals from each breed against the larger reference population, with HD genotypes masked, and the remaining 50k genotypes imputed to full density. Accuracy was then calculated for each dataset. Additionally, for the Romney animals within and across breed accuracy was also calculated, where 100 animals were imputed against a random sample of 500 of the remaining 516 purebred Romney, or 500 other animals comprised of the Coopworth, Corriedale, Perendale, Texel and Wiltshire breeds. To account for biases in imputation accuracy for SNP with low MAF, the correction proposed by Hayes et al. [39] was applied.

### 2.3. Genome-Wide Association Studies

Both the discovery and validation genome-wide association studies (GWAS) was performed within SNP & Variation Suite v8.4.0 (Golden Helix, Inc., Bozeman, MT, USA, www.goldenhelix.com (accessed on 20 March 2021)) using two methods. Firstly, an Efficient Mixed-Model Association eXpedited (EMMAX) analysis using a kinship matrix was performed on log-transformed serum GGT levels at 21 days after a measured sporidesmin challenge (GGT21). Contemporary group (incorporating flock, year of birth, sex and mob [30]) and the first two principal components, calculated from genotypic data (to account for population stratification), were fitted as additional covariates. Secondly, genotype association tests were performed on the residuals obtained from ASReml (v4.1) [40] after fitting recorded pedigree and the fixed effect of the contemporary group. Population stratification was again corrected by fitting the first two principal components, calculated from genotypic data, in the analysis. After Bonferonni correction, the threshold for genome-wide significance (*p* < 0.05) was 1.04 × 10^−7^ and 3.39 × 10^−6^ for discovery and validation analyses, respectively.

### 2.4. Validation of Markers of Interest

To calculate the proportion of variance explained by each marker, SNP effects were estimated in ASReml by fitting the SNP as a fixed effect along with the contemporary group. The predicted trait values were converted to genotypic effects as follows *a* = (AA − BB)/2 and *d* = AB − ((AA + BB)/2)), where AA, BB and AB are the predicted trait values for each genotype class. The proportion of additive genetic variance due to each SNP was estimated as (*2pq* (*a* + *d*(*q* − *p*))^2^)/*V_A_*, where *p* and *q* are the allelic frequencies at the SNP locus, and *V_A_* is the total additive genetic variance of the trait obtained when no SNP fixed effects are included in the model. 

To quantify the predictive ability of markers of interest, 5-fold cross-validation was performed with random sampling (*n* = 50 iterations) within SNP & Variation Suite v8.4.0 for genomic best linear unbiased prediction (GBLUP). Only animals that had been genotyped on either the Ovine Infinium^®^ HD SNP BeadChip or the Illumina OvineLD BeadChip (*n* = 2999) were used for the analysis, and fixed effect coefficients included contemporary group. K-fold cross-validation was run including all markers (*n* = 12,500), and after removing markers in the regions of interest on chromosomes 15 and 24 (*n* = 12,477). Accuracies were calculated by dividing the Pearson product-moment correlation coefficient by the square root of the heritability.

To test whether SNP markers were predictive of haemoglobin phenotype (Hb-A, Hb-AB or Hb-B), blood was collected from 12 animals with different genotypes in the β-globin locus, using SNPs rs405755938 and rs402069107 (Table 1). Proteomic analyses are described in Supplementary Methods (Methods S1). 

## 3. Results

### 3.1. Imputation

Imputation accuracy, corrected for MAF (IA_MAF_), was consistent among the datasets tested. Romney, which was the largest of the purebred groups (*n* = 616) had a marginally higher accuracy of 0.987, compared to the smaller group of purebred Perendale (*n* = 207; IA_MAF_ = 0.983). Primera (*n* = 149), which can be considered a composite breed and therefore more closely reflects the entire reference set, had the highest imputation accuracy of 0.989. When the 100 Romney animals were imputed against a reference of 500 of the remaining Romney, or 500 purebred animals (excluding Romney and composite breeds), IA_MAF_ reduced to 0.962 and 0.932, respectively.

### 3.2. Genome-Wide Association Studies

**Figure 1:**
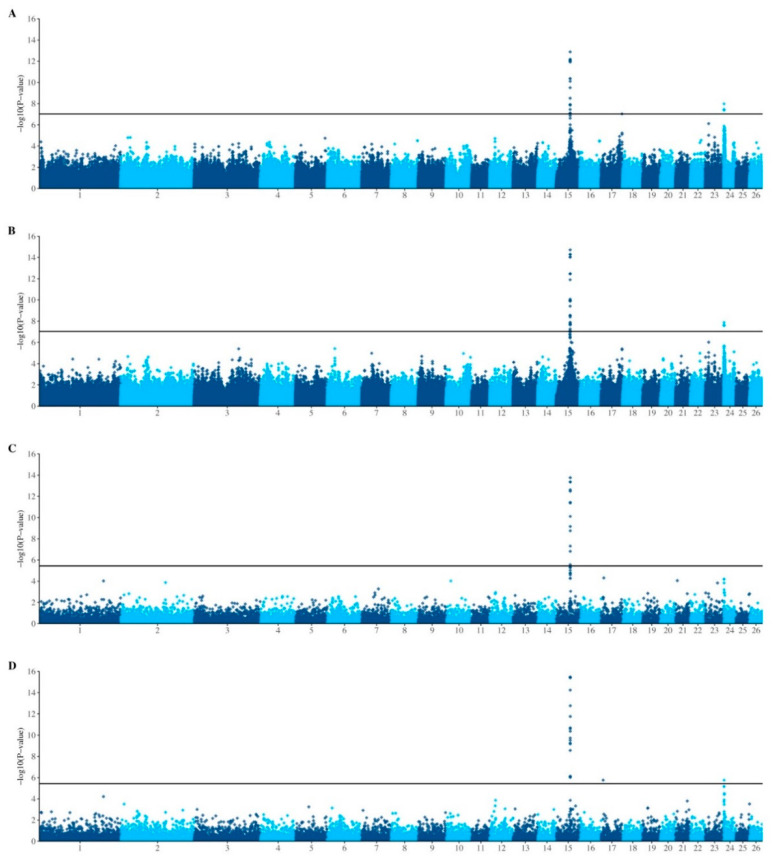
Manhattan plots of genome-wide association analysis for Facial Eczema in New Zealand sheep. Efficient Mixed-Model Association eXpedited (EMMAX) analyses using a kinship matrix (**A**,**C**) were conducted using log-transformed serum GGT levels at 21 days after a measured sporidesmin challenge (GGT21), with the contemporary group and the first two principal components fitted as covariates. Genotype association tests (**B**,**D**), fitting two principal components, were performed using residuals obtained from ASReml after fitting pedigree and the fixed effect of the contemporary group. Animals had either high-density (genotyped using either the Ovine Infinium^®^ HD SNP BeadChip or the Illumina OvineSNP50 BeadChip, and imputed to HD) (**A**,**B**) or low-density (Illumina OvineLD BeadChip) (**C**,**D**) genotypes. After Bonferonni correction, the threshold for genome-wide significance (*p* < 0.05) was 1.04 × 10^−7^ (log-transformed = 6.98) and 3.39 × 10^−6^ (log-transformed = 5.47) for high-density and low-density analyses, respectively.

**Figure 2:**
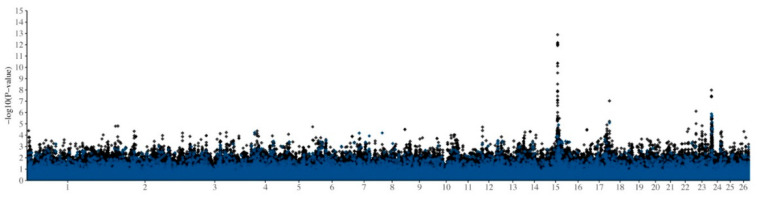
Comparison of imputed HD and 50K genotype data. Efficient Mixed-Model Association eXpedited (EMMAX) analysis of log-transformed serum GGT levels at 21 days after a measured sporidesmin challenge (GGT21), fitting contemporary group and the first two principal components fitted as covariates. Blue diamonds (♦) show *p*-values for the ~41k SNPs from the Illumina OvineSNP50 BeadChip. Black diamonds (♦) show *p*-values for the additional Ovine Infinium^®^ HD SNP BeadChip SNPs.

To investigate regions of the genome that are associated with FE, imputed HD genotype data were combined with log-transformed serum GGT levels at 21 days after a measured sporidesmin challenge (GGT21) for additive GWAS analysis (Figure 1A,B and Figure S1). In both EMMAX and association analyses a significant quantitative trait locus (QTL) was observed on chromosome 15, with a smaller peak apparent on chromosome 24. Significant SNPs from both QTL (17 SNPs on chromosome 15 and 5 SNPs on chromosome 24) were subsequently included in the design of the Illumina OvineLD BeadChip (Table S1). Figure 2 shows the EMMAX results from the discovery analysis, using genotypes that had been imputed from 50K to HD (600K). When these results are reduced to only the ~41k SNPs that are present on both BeadChips, no significant QTL were observed.

**Figure 3:**
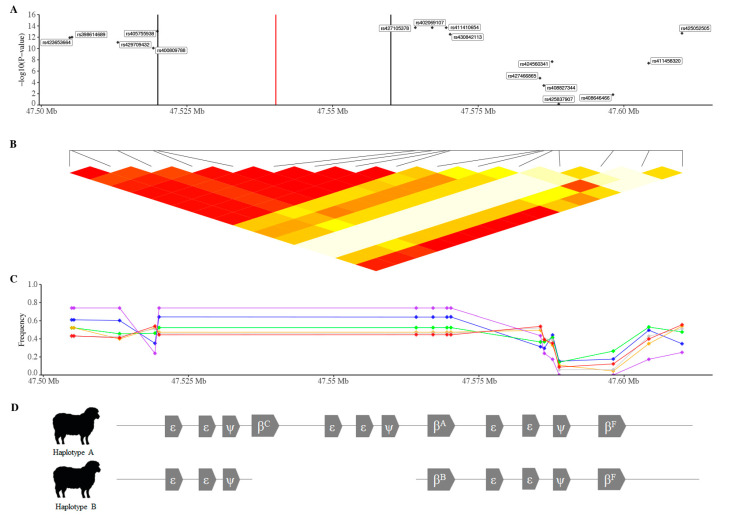
Detailed examination of the region underlying the GWAS peak on chromosome 15 (47,490 Kb–47,650 Kb). (**A**) Efficient Mixed-Model Association eXpedited (EMMAX) analysis using animals genotyped with either the Ovine Infinium^®^ HD SNP BeadChip or Illumina OvineLD BeadChip. (**B**) The previously reported [41] ~37-kb gap in the v3.1 sheep genome assembly is indicated by the red line. The ~40-kb region of low sequence identity between haplotype A and B (reference genome) is shown by the two black lines. Linkage disequilibrium (R^2^), from 1 (red) to 0 (white). (**C**) Allele frequency (based on overall minor allele) by breed: Composite (blue); Coopworth (green); Highlander (orange); Perendale (grey); Romney (red); Wiltshire (purple), based on overall minor allele. (**D**) Schematic diagram of the β-globin locus (duplication of an ancestral four-gene set, ε-ε-ψ-β), showing the arrangement of haplotype A and B. While both haplotypes A and B contain the adult β-globin gene, *HBB*, the two are considered allelic variants (β^A^ and β^B^).

Low-density genotyping (Illumina OvineLD BeadChip) of additional animals with FE phenotypes confirmed the QTL on chromosome 15, using both EMMAX and association analyses (Figure 1C,D). The SNPs in this region (Figure 2) are not present on the Illumina OvineSNP50 BeadChip, therefore only animals that had been genotyped on either the Ovine Infinium^®^ HD SNP BeadChip or the Illumina OvineLD BeadChip (*n* = 2999; 13,212 shared markers) were used to repeat the EMMAX analysis (Figure 3A), calculate linkage disequilibrium (R^2^) between markers (Figure 3B) and allele frequency by breed (Figure 3C).

### 3.3. Regions of Interest

A total of 11 SNPs, all on chromosome 15, reached genome-wide significance in all analyses (Figure 1 and Table 1). In addition, two SNPs on chromosome 24 reached genome-wide significance in both the high-density genotype analyses (Figure 1A,B) and the combined analysis (Table 1). Minor allele frequencies ranged from 0.33 to 0.5. Dominance effects were significant for all SNPs aside from rs398930318. The proportion of the phenotypic variance explained by individual SNPs ranged from 1% to 5%.

The significant region on chromosome 15 (15:47504887-47609978; Figure 3) has previously been identified as the location of the β-globin locus [41]. Of the significant SNPs in the region (Table 1), one (rs402069107) is a missense variant within the *HBB* gene, with an A > G change at position 359 of the coding sequence resulting in a change from Asparagine (N) to serine (S) at amino acid 120. In sheep, the repetitive β-globin locus has two different haplotypes, the longer haplotype A and the shorter haplotype B. While both haplotypes A and B contain the adult β-globin gene, *HBB*, the two are considered allelic variants (β^A^ and β^B^) [42,43] differing structurally by at least seven amino acids [44,45,46]. Version 3.1 of the ovine genome (OAR_v3.1), which all of the SNPs were designed from and mapped to, contains the shorter haplotype B [41]. There were, therefore, no SNPs on any of the genotyping platforms used in this study that were only present in the longer haplotype A.

The two SNPs immediately on either side of the β-globin region that uniquely mapped to multiple public genome assemblies (Table 2), rs405755938 and rs402069107, were used to estimate the β-globin haplotype. All haplotype A animals were called CC (the B allele in Table 1) at rs405755938, and GG (the B allele in Table 1) at rs402069107, with the haplotype B animals homozygous for the alternate allele (TT and AA, respectively). The rs402069107 alleles are consistent with HBBA (UniProt ID Q1KYZ7) and HBB (UniProt ID P02075) protein sequences; the G allele results in an S at amino acid 120 of the HBB protein, as seen in HBBA (β^A^). At both positions, the predicted GGT21 values from ASReml were lower for the haplotype A animals (Table 1).

The significant genome region on chromosome 24 is approximately 230 Kb long. This region reached significance in both high-density genotype analyses, however, only one SNP reached significance in the EMMAX analysis of the low-density genotypes, and this SNP was almost 300Kb upstream of the region identified by the high-density genotypes. Of the significant SNPs on chromosome 24 from the combined EMMAX analysis (Table 1), one (rs425270036) is a missense variant within the *PTX4* gene.

Including markers in the regions of interest from chromosomes 15 and 24 increased GBLUP accuracy from 0.491 to 0.505, although this was not significant (Table 3).

### 3.4. Proteomic Analysis

The A and B variants of sheep β-globin (Figure S2) were characterised by the following amino acid changes from A (UniProt ID: Q1KYZ7)→B (UniProt ID: P02075), respectively: S^59^→N, A^67^→P, VQ^75^→MK, S^119^→N, E^128^→D and R^143^→K. These amino acid changes are found in four tryptic peptides from haemoglobin β. In the A variant, these peptides are defined by the following residues: 40–58, 65–81, 116–131 and 132–143. In the B variant, the change of Q^75^→K results in a shorter peptide 65–75 but the other identifier tryptic peptides are of the same length. Following the LC-MS run, genotypes (rs405755938 and rs402069107) at the β-globin locus predicted β-globin peptides (β^A^, β^AB^ or β^B^) for 11 of the 12 animals, with one animal, predicted to be Hb-B, 5745, also showing polymorphism two of the seven variable peptides (Table 4).

## 4. Discussion

In this study, we used large phenotypic datasets in conjunction with high- and low-density genotype data to interrogate the sheep genome for regions associated with variability in tolerance to Facial Eczema (FE). Two QTL on chromosomes 15 and 24, were discovered using imputed high-density genotypes. The significant SNPs were subsequently included on a bespoke low-density ovine SNP chip for validation using industry-phenotyped animals. This study highlights the power of using increased density genotyping for the identification of influential genomic regions. SNPs from the Illumina OvineSNP50 BeadChip were not associated with the QTL on chromosome 15, likely due to the high levels of diversity in the breeds represented in this study, and the resulting reduced linkage disequilibrium between markers [48]. When the variants of importance are subsequently included on more widely used, lower density genotyping platforms, this enables validation in a larger number of animals at a lower cost, through alignment with industry-collected genotypes and phenotypes.

The 13 SNPs examined had largely additive effects, although there was some evidence for dominance. If these genotypes are also used in genomic selection, they may increase breeding value accuracy, although this is largely captured by GBLUP. Given the subclinical manifestation of the disease, further work is needed to understand the impact of these effects on the phenotype.

The significant region (*p* < 1.04 × 10^−7^) on chromosome 24 from the analysis using the high-density genotypes is approximately 230 Kb. Of the SNPs that reached significance in the high-density SNP analysis, two are missense variants within the *PTX4* gene. *PTX4* is reported to be widely expressed at low levels, with the highest levels in the liver in mice, and the small intestine and testes in humans [49], however, expression in sheep has been detected primarily in the cerebellum, at low levels [37,50]. *PTX4* belongs to the pentraxin (PTX) superfamily of multifunctional conserved proteins; some PTXs are part of the humoral arm of innate immunity and behave as functional ancestors of antibodies by mediating agglutination and complement activation, however, *PTX4* mRNA expression is distinct from other PTX family members [49], and there is no current evidence supporting the function of *PTX4* in innate immunity [51].

The significant region on chromosome 15 contains the β-globin locus. In ruminants, the β-globin locus was formed by the duplication of an ancestral four-gene set, ε-ε-ψ-β, consisting of two embryonic-like genes (ε), a pseudogene (ψ) and a β (β) globin gene [52]. Goats have three of the four-gene sets, where each set contains a different form of the β-globin gene. The three β-globin proteins are synthesised to make different forms of haemoglobin during development; in the foetus (β^F^), pre-adult (β^C^) and the adult (β^A^) [53]. In cattle, the β-globin locus contains two four-gene sets, containing β^F^ and β^A^, with the foetal cluster containing an additional β-like pseudogene [54]. In sheep, the β-globin locus has two different haplotypes, A and B [42,43,55,56]. The arrangement of haplotype A is identical to that of the goat, containing three four-gene sets, whereas haplotype B, like cattle, lacks the four-gene set containing the pre-adult β^C^. While both haplotypes A and B contain the adult β-globin gene, *HBB*, the two are considered allelic variants (β^A^ and β^B^) [42,43] differing structurally by at least seven amino acids [44,45,46]. Haemoglobin (Hb) from adult sheep with haplotype A (Hb-A; α_2_β_2_^A^) and B (Hb-B; α_2_β_2_^B^) is distinguishable electrophoretically [57].

The OARv3.1 (GCA_000298735.2) sheep genome contains the shorter haplotype B (Table 2), either as a result of the phenotypes of animals selected for sequencing or the challenges faced when assembling repetitive regions of the genome using short-read sequencing [58]. In comparison, the Rambouillet genome (ARS-UI_Ramb_v2.0; GCA_016772045.1), created using HiSeq X Ten and PacBio RS II reads, contains the longer haplotype A. There are several SNPs in the β-globin region on the high-density chip that appear to be copy number variants (CNVs) in animals with the longer haplotype A, mapping uniquely to OARv3.1 but mapping to two locations in both Oar_rambouillet_v1.0 (GCA_002742125.1) and ARS-UI_Ramb_v2.0 (Table S1). The Rambouillet genome should therefore also be used for future work searching for more accurate genetic markers for haemoglobin type in New Zealand sheep.

Various studies have shown variation in the frequency of haemoglobin A and B, indicating that there are advantages to both types [59]. Hb-A has a higher affinity for molecular oxygen than Hb-B and is, therefore, less efficient in transporting oxygen as it releases oxygen at a lower rate than normal [56,60,61,62]. As a result, Hb-B sheep tolerate anaemia better than Hb-A sheep due to their greater ability to liberate oxygen to tissue [56,59,60], whereas Hb-A sheep are more resistant to hypoxia [63]. Adult haplotype A sheep can switch from the synthesis of Hb-A (α_2_β_2_^A^) to the juvenile haemoglobin C (Hb-C; α_2_β_2_^C^) in response to hypoxia [63,64], anaemia [65,66,67] or erythropoietin injection [68]. It has been suggested that Hb-C is advantageous in compensating for relative tissue hypoxia of sheep and goats during severe anaemic states [69]. The β-globin locus is introgressed into Tibetan sheep from argali (*Ovis ammon*), suggesting the importance of the locus for sheep living in high altitude and low oxygen environments [70].

Our results indicate that the β-globin locus haplotype, and therefore ovine haemoglobin type, can be estimated using SNPs within the β-globin locus, although one animal that was hypothesised to be homozygous for haplotype B (β^B^) appeared to also have a shortened form of the β^A^-globin peptide. It appears that haplotype A (haemoglobin A; Hb-A) animals are more tolerant to FE. The overall frequency of haplotype A in the animals genotyped in this study, however, is 0.5, indicating that the locus may be under balancing selection in these populations, and warrants further investigation. In crossbred ewes, Hb-A has been associated with beneficial effects for several health-related traits, including mastitis and parasite resistance, whereas Hb-B was associated with increased fertility [71]. In Norway, Hb-A lambs have been reported to be significantly more resistant to hepatogenous photosensitisation (alveld) as a result of ingestion of *Nathercium ossifragum* than Hb-B animals [72]. Hepatogenous photosensitisation, known as geeldikkop, also occurs in small ruminants grazing *Tribulus terrestris* in South Africa. While the pathogenetic mechanisms underlying geeldikkop and FE differ, both affect the biliary tree, resulting in the accumulation of phylloerythrin [73]. In agreement with this study, and the Norwegian studies, Hb-A was shown to have a functional advantage in sheep affected by geeldikkop; both Hb-A and Hb-AB lambs were more resistant to serious haemolytic episodes, and a more severe temporary liver insufficiency developed in Hb-B animals than Hb-AB types [74]. Haemoglobin C (Hb-C) was present in Merino sheep recovering from a geeldikkop attack but was not present in the animals before the onset of the disease, with the authors suggesting that it may aid in recovery from the disease [75]. Of additional interest is that the frequency of haplotype A was higher in South African farms that had large scale outbreaks of haemolytic syndromes in their recent history [75]. Previous work looking at the distribution of haemoglobin in New Zealand sheep has found both types present in both Romney and Finnish Landrace breeds, with the A haplotype at a frequency of 0.6 and 0.84 in each breed, respectively [76]. Finnish Landrace lambs have also been reported to be less susceptible to a sporidesmin challenge than Romney lambs [77].

The underlying role of haemoglobin type in the response to FE is not straightforward. The mechanism of toxicity of sporidesmin, the causative mycotoxin, is believed to be through the generation of reactive oxygen species including superoxide radicals, leading to liver damage [78]. Other modes of action have also been postulated and may warrant further examination [79]. Antioxidants scavenge reactive oxygen species, and research in mice and human cell lines suggest haemoglobin may function as an antioxidant, suppressing oxidative stress and protecting cells from oxidative damage. Overexpression of haemoglobin in both murine kidney [80] and dopaminergic [81] cell lines impacted genes involved in oxidative stress and O_2_ homeostasis, respectively, and suppressed oxidative stress in human liver cell lines [82]. Antioxidant enzymes including superoxide dismutase (SOD), glutathione peroxidase (GPx) and catalase (CAT) have all been investigated as potential indicators of FE resistance. In the FE selection line animals, there was an overlap between enzyme activity distributions of the lines, therefore differences in enzyme activity were not large enough to be a reliable indicator of FE tolerance [83]. Analysis of allele frequencies in the FE selection line animals found significant differences between markers flanking the catalase gene (*CAT*) between the resistant and susceptible line, however, there was no evidence of linkage in outcross pedigrees, and it was concluded that the effect of catalase was probably recessive or minor [31]. Of interest is that the catalase gene is also located on chromosome 15, albeit 15.5 Mb from the β-globin locus. Additionally, a genome-scan experiment to screen for QTL affecting FE tolerance in Romney sheep identified a suggestive (F-statistic value > 10.2) QTL on chromosome 15 (23.4–73.8 Mbp), which, while large, overlaps the β-globin locus.

While the frequency of the haemoglobin haplotypes in the FE selection lines is unknown, in an analysis of 120 New Zealand Romney ewes of known haemoglobin type, Hb-B sheep had significantly higher levels of plasma copper, whole-blood selenium, and erythrocytic GPx than Hb-A sheep [76]. Previous studies also found Hb-B sheep had higher levels of plasma copper than type A sheep [84,85,86], and a strong positive relationship between copper and GGT levels post-exposure to sporidesmin has recently been reported [87]. A QTL for plasma copper concentration has been reported to be close to the haemoglobin locus in sheep [88]. The biological links between exposure to sporidesmin, haemoglobin type, antioxidant enzymes and copper do not appear to be straightforward and warrant further investigation.

## 5. Conclusions

Two loci for tolerance to facial eczema in sheep are reported on chromosomes 15 and 24. Markers at each locus explain 5% and 1% of the phenotypic variance in GGT21 levels after a sporidesmin challenge, respectively. The significant region on chromosome 15 overlaps the β-globin locus; mass spectrometry of haemoglobin from animals with differing genotypes in this region indicated that genotypes are associated with different forms of adult β-globin. Haplotype A animals appear to be more tolerant to FE. Given the importance of the β-globin region and observed intermediate haplotype frequencies of the animals in this study, the region may be under balancing selection. Further work is needed to explore this in a larger dataset. Haemoglobin haplotypes have previously been associated with variation in several health-related traits in sheep, and therefore warrant further investigation regarding their role in tolerance to facial eczema in sheep. This study highlights the power of using increased density genotyping for the identification of influential genomic regions, combined with subsequent inclusion on lower density genotyping platforms for rapid validation and industry uptake.

## Figures and Tables

**Table 1:** SNPs that reached genome-wide significance levels for Facial Eczema tolerance. EMMAX *p*-values, predicted log-transformed GGT21 values, allele substitution effects (α), dominance effects (*d*) and proportion of total additive (V_A_) and phenotypic (V_P_) variance explained by each marker.

RSID	Chr	Position ^1^	−log_10_ *p*-Value ^2^	MAF (Allele) ^3^	Predicted GGT21 Values ^4^			α	*d*	Prop V_A_	Prop V_P_
					AA (±SE)	AB (±SE)	BB (±SE)				
rs398930318	15	47,489,709	6.09	0.45 (A)	5.04 (0.07)	4.9 (0.06)	4.63 (0.07)	−0.21	0.07	0.04	0.02
rs423653664	15	47,504,887	11.93	0.48 (A)	5.09 (0.07)	4.92 (0.06)	4.54 (0.07)	−0.28	0.10	0.07	0.04
rs398614689	15	47,505,272	12.00	0.48 (A)	5.09 (0.07)	4.92 (0.06)	4.54 (0.07)	−0.28	0.11	0.07	0.04
rs429709432	15	47,513,135	11.09	0.46 (A)	5.08 (0.07)	4.96 (0.06)	4.51 (0.07)	−0.29	0.16	0.08	0.04
rs400809788	15	47,519,231	10.07	0.49 (B)	5.09 (0.07)	4.94 (0.06)	4.49 (0.07)	0.30	0.14	0.08	0.04
rs405755938	15	47,519,931	13.07	0.50 (A)	5.09 (0.07)	4.92 (0.06)	4.49 (0.07)	−0.30	0.13	0.08	0.05
rs427105378	15	47,564,204	13.69	0.50 (B)	4.49 (0.07)	4.92 (0.06)	5.09 (0.07)	0.30	0.13	0.08	0.05
rs402069107	15	47,567,105	13.69	0.50 (A)	5.09 (0.07)	4.92 (0.06)	4.49 (0.07)	−0.30	0.13	0.08	0.05
rs411410654	15	47,569,499	13.69	0.50 (B)	4.49 (0.07)	4.92 (0.06)	5.09 (0.07)	0.30	0.13	0.08	0.05
rs430842113	15	47,570,178	12.51	0.50 (A)	5.10 (0.07)	4.94 (0.06)	4.50 (0.07)	−0.30	0.14	0.08	0.05
rs425052505	15	47,609,978	12.70	0.50 (B)	5.09 (0.07)	4.91 (0.06)	4.48 (0.07)	0.30	0.12	0.09	0.05
rs425270036	24	1,155,234	5.70	0.33 (B)	4.94 (0.06)	4.83 (0.06)	4.56 (0.08)	0.19	0.08	0.04	0.01
rs409675199	24	1,156,263	5.76	0.33 (A)	4.56 (0.08)	4.83 (0.06)	4.94 (0.06)	−0.19	0.08	0.04	0.01

^1^ Position is based on OARv3.1. ^2^ Combined EMMAX analysis using animals genotyped on either the Ovine Infinium^®^ HD SNP BeadChip or the Illumina OvineLD BeadChip (*n* = 2999; 13,212 shared markers). After Bonferonni correction, the threshold for genome-wide significance (*p* < 0.05) was 3.78 × 10^−6^ (log-transformed = 5.42). ^3^ Minor allele frequencies (MAF), with minor allele shown in brackets. Alleles are shown in AB format. ^4^ Predicted log-transformed serum GGT levels at 21 days after a measured sporidesmin challenge (GGT21) values estimated using ASReml.

**Table 2:** Genomic position of markers used to predict β-globin haplotype.

Genome Assembly	Genbank Accession	Chromosome	rs405755938	rs402069107	Region Distance
OAR_v3.1	GCA_000298735.2	15	47,519,931	47,567,105	65,291
Oar_v4.0	GCA_000298735.2	15	47,414,827	47,462,139	65,429
Oar_rambouillet_v1.0	GCA_002742125.1	15	51,898,128	51,984,579	104,993
ARS-UI_Ramb_v2.0	GCA_016772045.1	15	47,951,894	48,038,330	104,963
White Dorper ^1^		15	47,750,476	47,797,960	65,855
Romanov ^1^		15	48,107,973	48,194,400	104,969

^1^ Ben Rosen, pers. comm. [47].

**Table 3:** Summary statistics from K-fold (K = 5) cross-validation with random sampling (*n* = 50 iterations).

Markers	Fixed Effects ^1^	PPMCC ^2^ (± SD)	h^2^	GBLUP Accuracy ^3^
All SNPs (*n* = 12,500)	Contemporary group	0.335 ± 0.007	0.44	0.505
Removing markers in regions of interest (*n* = 12,477)	Contemporary group	0.326 ± 0.007		0.491

^1^ Contemporary group (incorporating flock, year of birth, sex and mob) were fitted as covariates in all analyses. ^2^ Pearson’s Product-Moment Correlation Coefficient (PPMCC). ^3^ GBLUP accuracy calculated as PPMCC/√h^2^.

**Table 4:** Unique β-globin peptides detected by mass spectrometry from sheep blood samples from genotyped animals.

Predicted β-Globin Type ^1^	β^A^	β^B^
β^A^ (*n =* 3)	001 MLTAEEKAAV TGFWGKVKVD EVGAEALGRL LVVYPWTQRF ********** ********** ********** ********** 041 FEHFGDLSSA DAVMNNAKVK AHGKKVLDSF SNGVQHLDDL ******** * ****** *** ********** *** ***** 081 KGTFAQLSEL HCDKLHVDPE NFRLLGNVLV VVLARHHGSE ********** ********** ********** ******** * 121 FTPVLQAEFQ KVVAGVANAL AHRYH ******* ** ********** ** **	
β^AB^ (*n =* 3)	001 MLTAEEKAAV TGFWGKVKVD EVGAEALGRL LVVYPWTQRF ********** ********** ********** ********** 041 FEHFGDLSSA DAVMNNAKVK AHGKKVLDSF SNGVQHLDDL ******** * ****** *** ********** *** ***** 081 KGTFAQLSEL HCDKLHVDPE NFRLLGNVLV VVLARHHGSE ********** ********** ********** ******** * 121 FTPVLQAEFQ KVVAGVANAL AHRYH ******* ** ********** ** **	001 MLTAEEKAAV TGFWGKVKVD EVGAEALGRL LVVYPWTQRF ********** ********** ********** ********** 041 FEHFGDLSNA DAVMNNPKVK AHGKKVLDSF SNGMKHLDDL ******** * ****** *** ********** *** ***** 081 KGTFAQLSEL HCDKLHVDPE NFRLLGNVLV VVLARHHGNE ********** ********** ********** ******** * 121 FTPVLQADFQ KVVAGVANAL AHKYH ******* ** ********** ** **
β^B^ (*n =* 1)	001 MLTAEEKAAV TGFWGKVKVD EVGAEALGRL LVVYPWTQRF ********** ********** ********** ********** 041 FEHFGDLSSA DAVMNNAKVK ******** * ****** ***	001 MLTAEEKAAV TGFWGKVKVD EVGAEALGRL LVVYPWTQRF ********** ********** ********** ********** 041 FEHFGDLSNA DAVMNNPKVK AHGKKVLDSF SNGMKHLDDL ******** * ****** *** ********** *** ***** 081 KGTFAQLSEL HCDKLHVDPE NFRLLGNVLV VVLARHHGNE ********** ********** ********** ******** * 121 FTPVLQADFQ KVVAGVANAL AHKYH ******* ** ********** ** **
β^B^ (*n =* 2)		001 MLTAEEKAAV TGFWGKVKVD EVGAEALGRL LVVYPWTQRF ********** ********** ********** ********** 041 FEHFGDLSNA DAVMNNPKVK AHGKKVLDSF SNGMKHLDDL ******** * ****** *** ********** *** ***** 081 KGTFAQLSEL HCDKLHVDPE NFRLLGNVLV VVLARHHGNE ********** ********** ********** ******** * 121 FTPVLQADFQ KVVAGVANAL AHKYH ******* ** ********** ** **

^1^ Predicted using genotypes from rs405755938 and rs402069107 (Table 1). β-globin sequence taken from UniProt (A: Q1KYZ7; B: P02075), consensus peptides between the two protein sequences are denoted by *.

## Data Availability

The data used in this study are in the Sheep Improvement Limited (https://www.sil.co.nz/ (accessed on 20 March 2021)) database. These data were collected from commercial sheep and thus the information is accessible only with permission from the flock owners.

## Supplementary Materials

The following are available online at https://www.mdpi.com/article/10.3390/genes12101560/s1, Table S1: FE discovery GWAS SNPs included in the design of the Illumina OvineLD BeadChip, mapped to publicly available ovine genome assemblies, Methods S1: Proteomic analysis of blood samples from genotyped animals, Figure S1: Quantile-quantile (QQ) plots of genome-wide association analysis for Facial Eczema in New Zealand sheep, Figure S2: Separation of haemoglobins from sheep blood by 4–20%T Tris-Tricine SDS-PAGE.

## References

[1] Di Menna M.E., Smith B.L., Miles C.O. (2009). A history of facial eczema (pithomycotoxicosis) research. N. Z. J. Agric. Res..

[2] Ariyawansa H.A., Hyde K.D., Jayasiri S.C., Buyck B., Chethana K.W.T., Dai D.Q., Dai Y.C., Daranagama D.A., Jayawardena R.S., Lücking R. (2015). Fungal diversity notes 111–252—taxonomic and phylogenetic contributions to fungal taxa. Fungal Divers..

[3] Smith B.L., Towers N.R. (2002). Mycotoxicoses of grazing animals in New Zealand. N. Z. Vet. J..

[4] Towers N.R. (2006). Mycotoxin poisoning in grazing livestock in New Zealand. Proc. N. Z. Soc. Anim. Prod..

[5] Smith B.L. (2000). Effects of low dose rates of sporidesmin given orally to sheep. N. Z. Vet. J..

[6] Moore R.W., Sumner R.M.W., Bass J.J., Hockey H.-U.P. (1983). Hogget lambing and its effect on the subsequent two-tooth performance of three breeds. Proc. N. Z. Soc. Anim. Prod..

[7] McMillan W.H., Dockrill G., Towers N.R. (1988). Sporidesmin poisoning in ewes during late pregnancy. Proc. N. Z. Soc. Anim. Prod..

[8] Morris C.A., Towers N.R., Wesselink C., Southey B.R. (1991). Effects of facial eczema on ewe reproduction and postnatal lamb survival in Romney sheep. N. Z. J. Agric. Res..

[9] Smith B.L., Towers N.R. Pithomycotoxicosis (facial eczema) in New Zealand and the use of zinc salts for its prevention. Proceedings of the Australia–USA Poisonous Plants Symposium.

[10] Beef + Lamb New Zealand (2019). Facing up to Facial Eczema.

[11] Clare N.T. (1944). Photosensitivity diseases in New Zealand. 3. The photosensitizing agent in facial eczema. N. Z. J. Sci. Technol..

[12] Flynn D.M. (1962). Facial eczema. 1. History of the disease in Victoria. J. Agric. Vic. Dep. Agric..

[13] Marasas W.F., Adelaar T.F., Kellerman T.S., Minné J.A., Van Rensburg I.B., Burroughs G.W. (1972). First report of facial eczema in sheep in South Africa. Onderstepoort J. Vet. Res..

[14] Brewer D., Russell D.W., de Melio Amaral R.E., Aycardi E.R. (1989). An examination of North and South American isolates of Pithomyces chartarum for production of sporidesmin and sporidesmolides. Proc. Nova Scotian Inst. Sci..

[15] Hansen D.E., McCoy R.D., Hedstrom O.R., Snyder S.P., Ballerstedt P.B. (1994). Photosensitization associated with exposure to Pithomyces chartarum in lambs. J. Am. Vet. Med. Assoc..

[16] Bezille P., Braun J.P., Le Bars J. (1984). First identification of facial eczema in Europe. Epidemiological, clinical and biological aspects. Rec. Med. Vet. Ec. Alfort..

[17] Liu L., Zhang Y., Sun Z., Ou K., Sun H., Yan G. (2014). Isolation and PCR-DGGE studies of the fungal pathogens from pastures with high incidence of sheep facial eczema on the northern slope of Tianshan Mountains. Acta Vet. Zootech. Sin..

[18] Brook P.J. (1963). Ecology of the fungus Pithomyces chartarum (Berk. & Curt.) M. B. Ellis in pasture in relation to facial eczema disease of sheep. N. Z. J. Agric. Res..

[19] Mitchell K.J., Walshe T.O., Robertson N.G. (1959). Weather Conditions Associated with Outbreaks of Facial Eczema. N. Z. J. Agric. Res..

[20] di Menna M.E., Bailey J.R. (1973). Pithomyces chartarum spore counts in pasture. N. Z. J. Agric. Res..

[21] Dennis N.A., Amer P.R., Meier S. (2014). BRIEF COMMUNICATION: Predicting the impact of climate change on the risk of facial eczema outbreaks throughout New Zealand. Proc. N. Z. Soc. Anim. Prod..

[22] Smith B.L., Embling P.P., Towers N.R., Wright D.E., Payne E. (1977). The protective effect of zinc sulphate in experimental sporidesmin poisoning of sheep. N. Z. Vet. J..

[23] Parle J.N., di Menna M.E. (1972). Fungicides and the control of Pithomyces chartarum. N. Z. J. Agric. Res..

[24] Towers N.R. (1986). Facial Eczema—Problems and Successes in Control. Proc. N. Z. Grassl. Assoc..

[25] Morris C.A., Towers N.R., Campbell A.G., Meyer H.H., Wesselink C., Wheeler M. (1989). Responses achieved in Romney flocks selected for or against susceptibility to facial eczema, 1975–1987. N. Z. J. Agric. Res..

[26] Campbell A.G., Meyer H.H., Henderson H.V., Wesselink C. (1981). Breeding for Facial Eczema Resistance—A Progress Report. Proc. N. Z. Soc. Anim. Prod..

[27] Towers N.R., Stratton G.C. (1978). Serum gamma-glutamyltransferase as a measure of sporidesmin-induced liver damage in sheep. N. Z. Vet. J..

[28] Morris C.A., Towers N.R., Wheeler M., Wesselink C. (1995). Selection for or against facial eczema susceptibility in Romney sheep, as monitored by serum concentrations of a liver enzyme. N. Z. J. Agric. Res..

[29] Amyes N.C., Hawkes A.D. (2014). Others Ramguard-increasing the tolerance to facial eczema in New Zealand sheep. Proc. N. Z. Soc. Anim. Prod..

[30] McRae K.M., Cullen N.G., Amyes N.C., Johnson P.L. (2016). Brief Communication: An update on genetic parameters for facial eczema tolerance in sheep. Proc. N. Z. Soc. Anim. Prod..

[31] Phua S.H., Dodds K.G., Morris C.A., Paterson K.A., McEwan J.C., Garmonsway H.G., Towers N.R., Crawford A.M. (1999). Catalase gene is associated with facial eczema disease resistance in sheep. Anim. Genet..

[32] Duncan E.J., Dodds K.G., Henry H.M., Thompson M.P., Phua S.H. (2007). Cloning, mapping and association studies of the ovine ABCG2 gene with facial eczema disease in sheep. Anim. Genet..

[33] Phua S.H., Dodds K.G., Morris C.A., Henry H.M., Beattie A.E., Garmonsway H.G., Towers N.R., Crawford A.M. (2009). A genome-screen experiment to detect quantitative trait loci affecting resistance to facial eczema disease in sheep. Anim. Genet..

[34] Phua S.H., Dodds K.G. Others Different methods detected different loci involved in resistance to facial eczema disease of sheep. Proceedings of the 19th Conference of the Association for the Advancement of Animal Breeding and Genetics.

[35] Phua S.H., Brauning R., Baird H.J., Dodds K.G. (2014). Identifying chromosomal selection-sweep regions in facial eczema selection-line animals using an ovine 50K-SNP array. Anim. Genet..

[36] Phua S.H., Hyndman D.L., Baird H.J., Auvray B., McEwan J.C., Lee M.A., Dodds K.G. (2014). Towards genomic selection for facial eczema disease tolerance in the New Zealand sheep industry. Anim. Genet..

[37] Jiang Y., Xie M., Chen W., Talbot R., Maddox J.F., Faraut T., Wu C., Muzny D.M., Li Y., Zhang W. (2014). The sheep genome illuminates biology of the rumen and lipid metabolism. Science.

[38] Sargolzaei M., Chesnais J.P., Schenkel F.S. (2014). A new approach for efficient genotype imputation using information from relatives. BMC Genom..

[39] Hayes B.J., Bowman P.J., Daetwyler H.D., Kijas J.W., van der Werf J.H.J. (2012). Accuracy of genotype imputation in sheep breeds. Anim. Genet..

[40] Gilmour A.R., Gogel B.J., Cullis B.R., Welham S.J., Thompson R. (2015). ASReml User Guide Release 4.1 Structural Specification.

[41] Jiang Y., Wang X., Kijas J.W., Dalrymple B.P. (2015). β-globin gene evolution in the ruminants: Evidence for an ancient origin of sheep haplotype B. Anim. Genet..

[42] Garner K.J., Lingrel J.B. (1988). Structural organization of the β-globin locus of B-haplotype sheep. Mol. Biol. Evol..

[43] Garner K.J., Lingrel J.B. (1989). A comparison of the β A-and β B-globin gene clusters of sheep. J. Mol. Evol..

[44] Boyer S.H., Hathaway P., Pascasio F., Orton C., Bordley J., Naughton M.A. (1966). Hemoglobins in sheep: Multiple differences in amino acid sequences of three β-chains and possible origins. Science.

[45] Wilson J.B., Edwards W.C., McDaniel M., Dobbs M.M., Huisman T.H.J. (1966). The structure of sheep hemoglobins. II. The amino acid composition of the tryptic peptides of the non-α chains of hemoglobins A, B, C, and F. Arch. Biochem. Biophys..

[46] Boyer S.H., Hathaway P., Pascasio F., Bordley J., Orton C., Naughton M.A. (1967). Differences in the amino acid sequences of tryptic peptides from three sheep hemoglobin β chains. J. Biol. Chem..

[47] Rosen B.D. Personal Communication, 2021.

[48] Kijas J.W., Porto-Neto L., Dominik S., Reverter A., Bunch R., McCulloch R., Hayes B.J., Brauning R., McEwan J. (2014). International Sheep Genomics Consortium Linkage disequilibrium over short physical distances measured in sheep using a high-density SNP chip. Anim. Genet..

[49] Martinez de la Torre Y., Fabbri M., Jaillon S., Bastone A., Nebuloni M., Vecchi A., Mantovani A., Garlanda C. (2010). Evolution of the pentraxin family: The new entry PTX4. J. Immunol..

[50] Clark E.L., Bush S.J., McCulloch M.E.B., Farquhar I.L., Young R., Lefevre L., Pridans C., Tsang H.G., Wu C., Afrasiabi C. (2017). A high resolution atlas of gene expression in the domestic sheep (*Ovis aries*). PLoS Genet..

[51] Wang Z., Wang X., Zou H., Dai Z., Feng S., Zhang M., Xiao G., Liu Z., Cheng Q. (2020). The Basic Characteristics of the Pentraxin Family and Their Functions in Tumor Progression. Front. Immunol..

[52] Townes T.M., Fitzgerald M.C., Lingrel J.B. (1984). Triplication of a four-gene set during evolution of the goat β-globin locus produced three genes now expressed differentially during development. Proc. Natl. Acad. Sci. USA.

[53] Schon E.A., Cleary M.L., Haynes J.R., Lingrel J.B. (1981). Structure and evolution of goat γ-, β C- and β A-globin genes: Three developmentally regulated genes contain inserted elements. Cell.

[54] Schimenti J.C., Duncan C.H. (1985). Structure and organization of the bovine β-globin genes. Mol. Biol. Evol..

[55] Cohen B.L., Evans J.V., Harris H., King J.W., Warren F.L. (1956). Genetics of haemoglobin and blood potassium differences in sheep. Nature.

[56] Huisman T.H., Van V., Sebens T. (1958). Some genetic and physiological aspects of two different adult haemoglobins in sheep. Nature.

[57] Harris H., Warren F.L. (1955). Occurrence of electrophoretically distinct haemoglobins in ruminants. Biochem. J.

[58] Treangen T.J., Salzberg S.L. (2011). Repetitive DNA and next-generation sequencing: Computational challenges and solutions. Nat. Rev. Genet..

[59] Dawson T.J., Evans J.V. (1967). Effect of anaemia on exygen transport in sheep with different haemoglobin types. Aust. J. Exp. Biol. Med. Sci..

[60] Dawson T.J., Evans J.V. (1962). Haemoglobin and Erythrocyte Potassium Types in Sheep and their Influence on Oxygen Dissociation and Haemoglobin Denaturation. Aust. J. Bio. Sci..

[61] Dawson T.J., Evans J.V. (1965). Effect of hemoglobin type on the cardiorespiratory system of sheep. Am. J. Physiol..

[62] Manca L., Pirastru M., Mereu P., Multineddu C., Olianas A., el Sherbini E.S., Franceschi P., Pellegrini M., Masala B. (2006). Barbary sheep (*Ammotragus lervia*): The structure of the adult β-globin gene and the functional properties of its hemoglobin. Comp. Biochem. Physiol. B Biochem. Mol. Biol..

[63] Dawson T.J., Evans J.V. (1966). Effect hypoxia on oxygen transport in sheep with different hemoglobin types. Am. J. Physiol..

[64] Battaglia F.C., Behrman R.E., De Lannoy C.W., Hathaway W., Makowski E.L., Meschia G., Seeds A.E., Schruefer J.J.P. (1969). Exposure to high altitude of sheep with different hæmoglobins. Q. J. Exp. Physiol. Cogn. Med. Sci..

[65] Blunt M.H., Evans J.V. (1963). Changes in the concentration of potassium in the erythrocytes and in hæmoglobin type in merino sheep under a severe anæmic stress. Nature.

[66] van Vliet G., Huisman T.H. (1964). Changes in the haemoglobin types of sheep as a response to anaemia. Biochem. J.

[67] Huisman T.H. (1974). The in vivo production of hemoglobin C in ruminants. Ann. N. Y. Acad. Sci..

[68] Huisman T.H., Lewis J.P., Blunt M.H., Adams H.R., Miller A., Dozy A.M., Boyd E.M. (1969). Hemoglobin C in newborn sheep and goats: A possible explanation for its function and biosynthesis. Pediatr. Res..

[69] Huisman T.H., Kitchens J. (1968). Oxygen equilibria studies of the hemoglobins from normal and anemic sheep and goats. Am. J. Physiol..

[70] Hu X.-J., Yang J., Xie X.-L., Lv F.-H., Cao Y.-H., Li W.-R., Liu M.-J., Wang Y.-T., Li J.-Q., Liu Y.-G. (2019). The Genome Landscape of Tibetan Sheep Reveals Adaptive Introgression from Argali and the History of Early Human Settlements on the Qinghai-Tibetan Plateau. Mol. Biol. Evol..

[71] Dally M.R., Hohenboken W., Thomas D.L., Craig A.M. (1980). Relationships between hemoglobin type and reproduction, lamb, wool and milk production and health-related traits in crossbred ewes. J. Anim. Sci..

[72] Laksesvela B., Dishington I.W. (1983). Bog asphodel (*Narthecium ossifragum*) as a cause of photosensitisation in lambs in Norway. Vet. Rec..

[73] Coetzer J.A., Kellerman T.S., Sadler W., Bath G.F. (1983). Photosensitivity in South Africa. V. A comparative study of the pathology of the ovine hepatogenous photosensitivity diseases, facial eczema and geeldikkop (*Tribulosis ovis*), with special reference to their pathogenesis. Onderstepoort J. Vet. Res..

[74] Neethling L.P., Brown J.M.M., Osterhoff D.R., De Wet P.J., Ward-Cox I.S. (1969). The functional advantage of haemoglobin type A in haemolytic syndromes in sheep. Phenylhydrazine, organic selenium and partial exsanguination as external agents in the production of anaemias. J. S. Afr. Vet. Assoc..

[75] Osterhoff D.R. (2009). Haemoglobin types and the geeldikkop-enzootic icterus disease complex in sheep. Anim. Blood Groups Biochem. Genet..

[76] Millar K.R. (1980). Haemoglobin types, blood parameters and erythrocyte glutathione in the New Zealand Romney and other breeds. N. Z. Vet. J..

[77] Morris C.A., Towers N.R., Wesselink C., Amyes N.C. (1994). Susceptibility of Finnish landrace, Romney, and Finn × Romney lambs to a sporidesmin challenge. N. Z. J. Agric. Res..

[78] Munday R. (1982). Studies on the mechanism of toxicity of the mycotoxin, sporidesmin. I. Generation of superoxide radical by sporidesmin. Chem. Biol. Interact..

[79] Jordan T.W. (2020). The cellular and molecular toxicity of sporidesmin. N. Z. Vet. J..

[80] Nishi H., Inagi R., Kato H., Tanemoto M., Kojima I., Son D., Fujita T., Nangaku M. (2008). Hemoglobin is expressed by mesangial cells and reduces oxidant stress. J. Am. Soc. Nephrol..

[81] Biagioli M., Pinto M., Cesselli D., Zaninello M., Lazarevic D., Roncaglia P., Simone R., Vlachouli C., Plessy C., Bertin N. (2009). Unexpected expression of α- and β-globin in mesencephalic dopaminergic neurons and glial cells. Proc. Natl. Acad. Sci. USA.

[82] Liu W., Baker S.S., Baker R.D., Nowak N.J., Zhu L. (2011). Upregulation of hemoglobin expression by oxidative stress in hepatocytes and its implication in nonalcoholic steatohepatitis. PLoS ONE.

[83] Hohenboken W.D., Morris C.A., Munday R., De Nicolo G., Amyes N.C., Towers N.R., Phua S.H. (2004). Antioxidants in blood from sheep lines divergently selected for facial eczema resistance. N. Z. J. Agric. Res..

[84] Wiener G., Hall J.G., Hayter S. (1973). An association between the concentration of copper in whole blood and haemoglobin type in sheep. Anim. Sci..

[85] Wiener G., Field A.C. (1974). Seasonal changes, breed differences and repeatability of plasma copper levels of sheep at pasture. J. Agric. Sci..

[86] Wiener G., Herbert J.G. (1976). Variation in liver and plasma copper concentrations of sheep in relation to breed and haemoglobin type. J. Comp. Pathol..

[87] Johnson P.L., Amyes N.C. (2020). An association between circulating copper concentrations and gammaglutamyl transferase activity in sheep after exposure to the toxin sporidesmin. N. Z. J. Anim. Sci. Prod..

[88] Neary D.M., Sutcliffe E., Haley C.S., Woolliams J.A. Single marker QTL mapping for copper in the plasma of sheep. Proceedings of the 6th World Congress on Genetics Applied to Livestock Production.

