# Supplementary material for: Genomic Tools for the Identification of Loci Associated with Facial Eczema in New Zealand Sheep
Source: Genes (Basel). 2021 Sep 30;12(10):1560. doi: 10.3390/genes12101560 (PMC8536008; doi:10.3390/genes12101560)
Supplement: Supplementary file 1 [file genes-12-01560-s001.zip › genes-1395896-supplementary.pdf]

Supplementary material

Supplementary Methods: Proteomic analysis of blood samples from genotyped animals

Animals were managed following the provisions of the New Zealand Animal Welfare Act 1999, and the New Zealand Codes of Welfare developed under sections 68–79 of the Act. The collection of blood samples was approved by the AgResearch Animal Ethics Committee (Application number 14842) and complied with the institutional Codes of Ethical Conduct for the Use of Animals in Research, Testing and Teaching, as prescribed in the New Zealand Animal Welfare Act of 1999 and its amendments.

To test whether SNP markers were predictive of haemoglobin phenotype (Hb-A, Hb-AB or Hb-B), blood was collected from 12 animals with different genotypes in the β-globin locus, using SNPs rs405755938 and rs402069107 (Table 1). Blood samples were obtained by jugular venipuncture and collected into 7-ml vacutainers containing EDTA as an anticoagulant.

Blood samples were centrifuged for 1,000 g for 20 mins at 4°C and the plasma removed. The red blood cells were then washed three times with cold 0.9% sodium chloride solution, the wash solution being removed by centrifugation at 1,000 g for 20 mins at 4°C. The cells were then lysed by gentle mixing with three-volume equivalents of 3.75 M phosphate buffer pH 7.2 at 4°C, then centrifuged at 19,000 g for 10 mins at 4°C. The supernatant was removed and stored at -20°C until further use.

Protein quantitation was performed on 20-times diluted samples with a Direct Detect Spectrometer (Millipore, Burlington MS, USA). 1 µg of each sample was then loaded onto a Criterion Tris-Tricine 4-20%T polyacrylamide gel in a Criterion Cell (Bio-Rad, Hercules, CA, USA) and separated by SDS-PAGE at 200 V, 80 mA and 15 W for 40 mins. The proteins were visualised by staining with Colloidal Coomassie Blue G250 (Plowman et al, 2010).

The protein bands were excised from the gel and destained by washing twice with 200 mM ammonium bicarbonate and 50% acetonitrile for 1 hr at 37°C. The bands were then reduced with a solution of 50 mM tris(2-carboxyethyl)phosphine in 100 mM ammonium bicarbonate at 56°C and then labelled with 150 mM iodoacetamide in 100 mM ammonium bicarbonate. The gel pieces were then digested overnight with 2 µg of TPCK-trypsin in 50 mM ammonium bicarbonate and 10% acetonitrile (AcN). Peptides were extracted from the gel by vortexing for 10 min in digestion buffer, followed by a solution of 50% AcN and then 80% AcN, all extracts being pooled with the original digestion supernatant. The extracts were then concentrated on a Centrivap vacuum centrifugal concentrator.

LC-MS was performed on a nanoflow Ultimate 3000 UPLC (Dionex) coupled to maXis Impact HD mass spectrometer equipped with a CaptiveSpray source (Bruker Daltonik, Bremen, Germany). For each sample, 1 µL of the sample was loaded on a C18 PepMap100 nano-Trap column (300 µm ID x 5 mm, 5 micron 100Å) at a flow rate of 3000 nL/min. The trap column was then switched in line with the analytical column ProntoSIL C18AQ (100 µm ID x 150 mm 3 micron 200Å). The reverse-phase elution gradient was from 2% to 20% to

45%B over 60 min, total 85 min at a flow rate of 600 nL/min. Solvent A was LCMS-grade water with 0.1% Formic acid; solvent B was LCMS-grade ACN with 0.1% FA.

The peptide digests were measured in positive ion MS mode with a mass range of m/z 350–1500 on the Impact HD mass spectrometer (Bruker Daltonik) in data-dependent MS/MS mode, with an acquisition speed of 2 Hz in MS and 1-20 Hz in MS/MS mode depending on precursor intensity. Ten precursors were selected in the m/z 350–1200 range, with a preference for doubly or triply charged peptides. The analysis was performed in positive ionization mode with a dynamic exclusion of 60 sec.

Following the LC-MS run, the QTOF data were searched using Peaks Studio 10.0 against a precursor mass tolerance of 10 ppm; fragment mass tolerance of 0.05 Da, a taxonomy of *Ovis aries* (with an in-house NCBI nr database), with trypsin as proteolytic enzyme and up to 2 miscleavages allowed. Carbamidomethyl (C) was specified as a fixed modification, and oxidation (M), deamidation (NQ) and phosphorylation (STY) were chosen as variable modifications. A false discovery rate (FDR) of 1% was used for peptide identification in Peaks. In addition, the Peaks post-translational modification (PTM) A-score was set to 50, *de novo* only ALC > 80% and only proteins with a minimum of 1 unique peptide identification were included.

The A and B variants of sheep β-globin were characterised by the following amino acid changes from A (UniProt ID: Q1KYZ7) → B (UniProt ID: P02075) respectively: S^{59} → N, A^{67} → P, VQ^{75} → MK, S^{119} → N, E^{128} → D and R^{143} → K. These amino acid changes are found in four tryptic peptides from haemoglobin β. In the A variant these peptides are defined by the following residues: 40-58, 65-81, 116-131 and 132-143. In the B variant, the change of Q^{75} → K results in a shorter peptide 65-75 but the other identifier tryptic peptides are of the same length. In SDS-PAGE sheep haemoglobin separates into two major bands around 15 kDa (Supplementary Figure 2).

Plowman, J.E., et al., *Characterisation of low abundance wool proteins through novel differential extraction techniques.* Electrophoresis, 2010. **31**(12): p. 1937-1946.

Table S1: FE discovery GWAS SNPs included in the design of the Illumina OvineLD BeadChip. Markers that mapped to more than one position are denoted with a *.

Assembly	OAR_v3.1		Oar_v4.0		Oar_rambouillet_v1.0		ARS-UI_Ramb_v2.0	
GenBank accession	GCA_000298735.2		GCA_000298735.2		GCA_002742125.1		GCA_016772045.1	
RSID	Chr.	Position	Chr.	Position	Chr.	Position	Chr.	Position
rs428441961*	15	47,425,386	15	47,318,992	15	51,796,047	15	47,849,872
rs420422985	15	47,453,268	15	47,346,818	15	51,823,702	15	47,877,515
rs418466829	15	47,458,507	15	47,352,057	15	51,828,912	15	47,882,722
rs426368387	15	47,473,013	15	47,367,857	15	51,843,345	15	47,897,154
rs398930318*	15	47,489,709	15	47,384,553	15	51,859,818	15	47,913,671
rs409516182*	15	47,494,685	15	47,389,529	15	51,872,554	15	47,926,390
rs423653664	15	47,504,887	15	47,399,783	15	51,882,659	15	47,936,440
rs398614689	15	47,505,272	15	47,400,168	15	65,525,783	15	47,936,821
rs429709432*	15	47,513,135	15	47,408,031	15	51,805,138	15	47,944,916
rs429709432*	-	-	-	-	15	51,891,091	-	-
rs400809788	15	47,519,231	15	47,414,127	15	51,897,388	15	47,951,156
rs405755938	15	47,519,931	15	47,414,827	15	51,898,128	15	47,951,894
rs428441961*	-	-	-	-	15	51,900,599	15	47,954,363
rs427105378*	15	47,564,204	15	47,459,238	15	51,981,666	15	48,035,417
rs402069107	15	47,567,105	15	47,462,139	15	51,984,579	15	48,038,330
rs411410654	15	47,569,499	15	47,464,533	15	51,986,973	15	48,040,724
rs430842113	15	47,570,178	15	47,465,212	15	51,987,652	15	48,041,403
rs408827344*	15	47,586,265	15	47,481,299	15	51,961,760	15	48,015,512
rs408827344*	-	-	-	-	15	52,004,953	15	48,058,696
rs427105378*	15	47,608,325	15	47,503,541	15	52,092,964	15	48,080,935
rs425052505	15	47,609,978	15	47,505,194	15	52,094,618	15	48,082,589
rs398930318*	-	-	-	-	15	65,510,363	-	-
rs409516182*	-	-	-	-	15	65,515,335	-	-
rs420122004	24	932,184	24	919,490	24	940,274	24	1,506,069
rs425270036	24	1,155,234	24	1,152,961	24	1,125,292	24	1,690,999
rs409675199	24	1,156,263	24	1,153,990	24	1,126,288	24	1,691,995
rs422823972	24	1,459,984	24	1,460,494	24	1,430,759	24	1,996,418
rs404562811	24	3,795,530	24	3,795,523	24	3,829,144	24	4,340,054

Figure S1: Quantile-quantile (QQ) plots of genome-wide association analysis for Facial Eczema in New Zealand sheep.

Efficient Mixed-Model Association eXpedited (EMMAX) analysis using a kinship matrix (A, C) were conducted using log-transformed serum GGT levels at 21 days after a measured sporidesmin challenge (GGT21), with the contemporary group and the first two principal components fitted as covariates. Genotype association tests (B, D), fitting two principal components, were performed using residuals obtained from ASReml after fitting pedigree ad the fixed effect of the contemporary group. Animals had either high-density (genotyped using either the Ovine Infinium® HD SNP BeadChip or the Illumina OvineSNP50 BeadChip, and imputed to HD) (A, B) or low-density (Illumina OvineLD BeadChip) (C, D) genotypes.

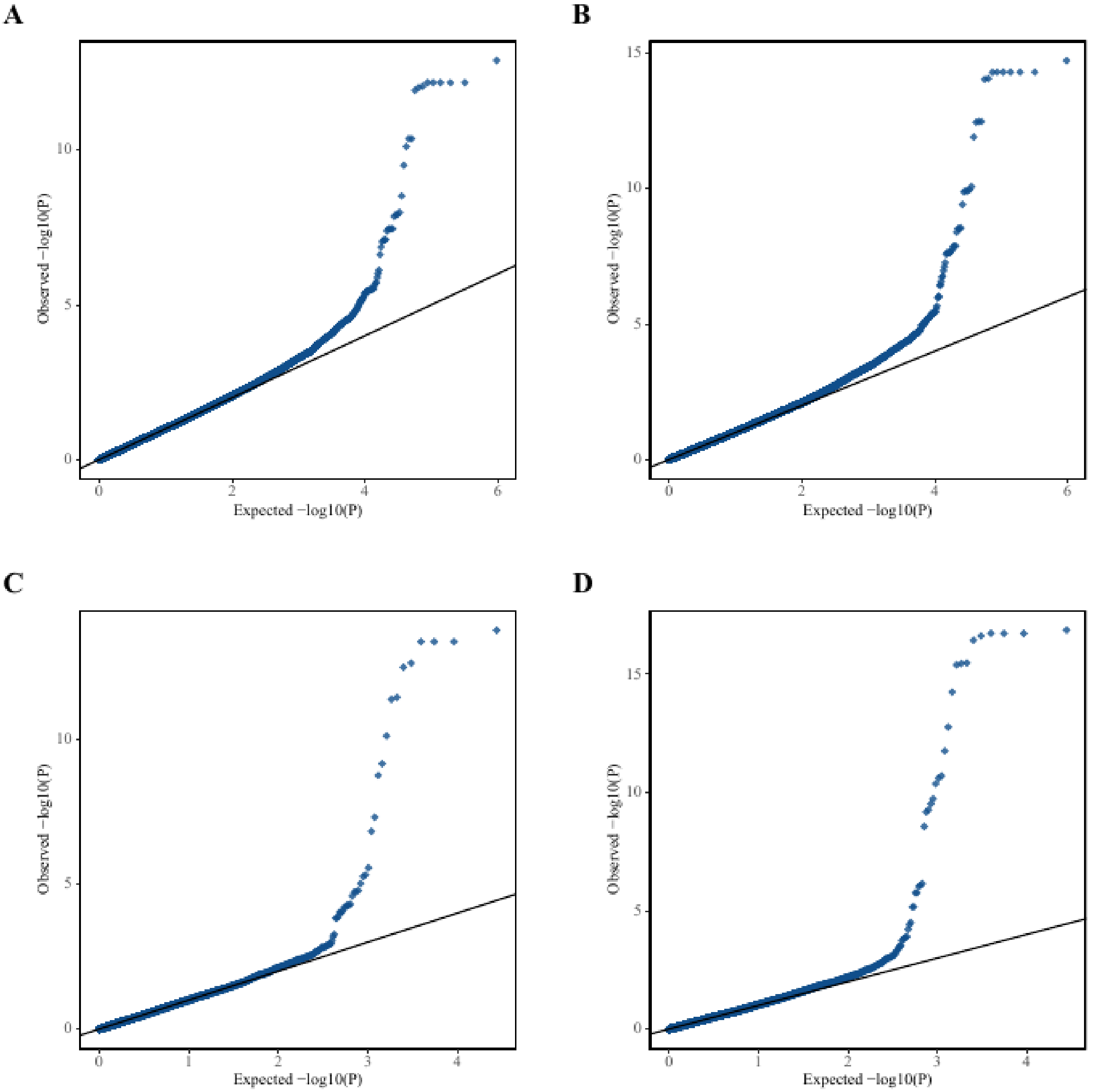

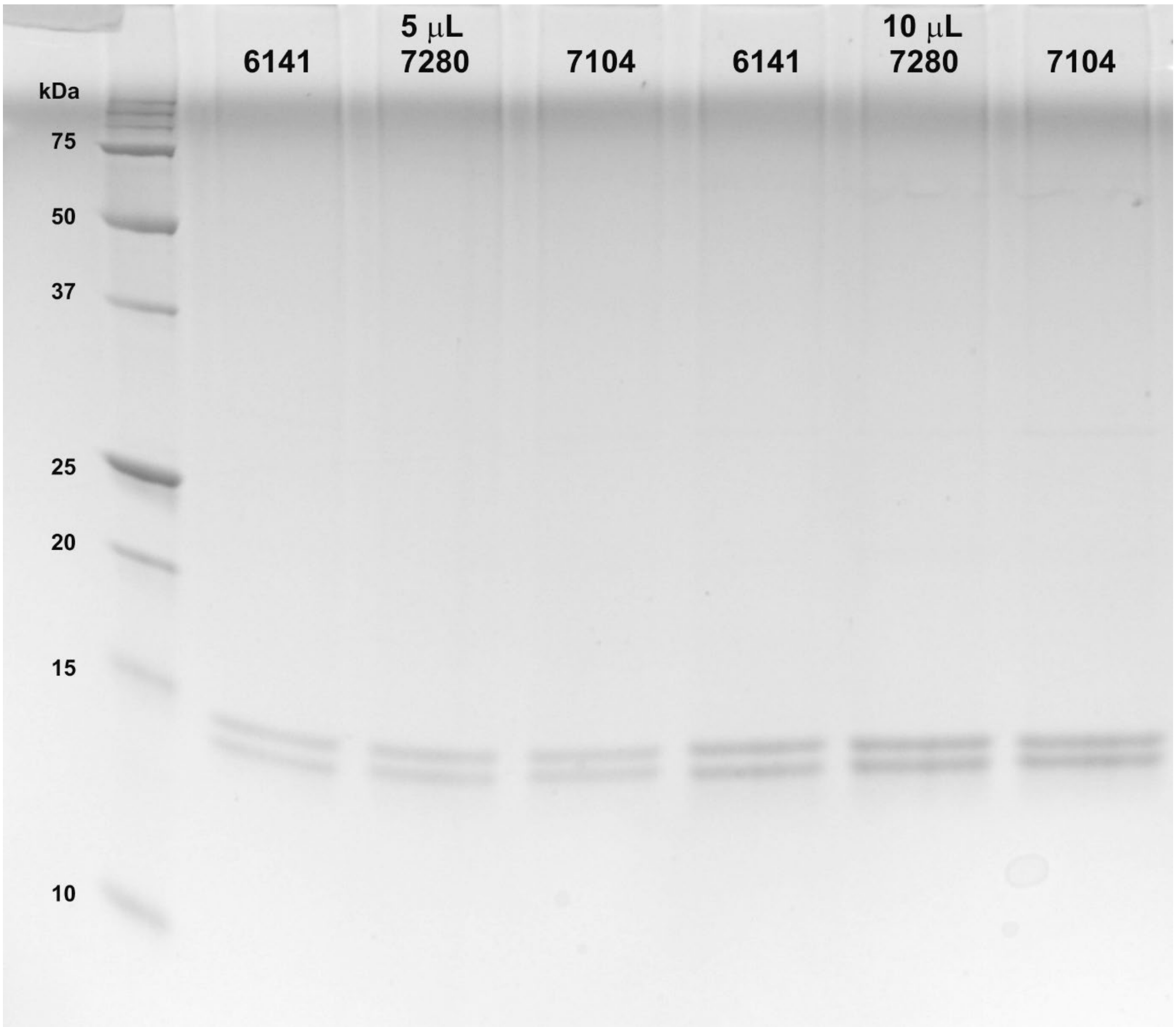

Figure S2: Separation of haemoglobins from sheep blood by 4-20%T Tris-Tricine SDS-PAGE.
